# Identification of novel M2 macrophage‐related molecule ATP6V1E1 and its biological role in hepatocellular carcinoma based on machine learning algorithms

**DOI:** 10.1111/jcmm.70072

**Published:** 2024-09-18

**Authors:** Sen Zhao, Meimei Liu, Hua Zhou

**Affiliations:** ^1^ School of Basic Medical Anhui Medical College Hefei Anhui China

## Abstract

Hepatocellular carcinoma (HCC) remains the most prevalent form of primary liver cancer, characterized by late detection and suboptimal response to current therapies. The tumour microenvironment, especially the role of M2 macrophages, is pivotal in the progression and prognosis of HCC. We applied the machine learning algorithm—CIBERSORT, to quantify cellular compositions within the HCC TME, focusing on M2 macrophages. Gene expression profiles were analysed to identify key molecules, with ATP6V1E1 as a primary focus. We employed Gene Set Enrichment Analysis (GSEA) and Kaplan–Meier survival analysis to investigate the molecular pathways and prognostic significance of ATP6V1E1. A prognostic model was developed using multivariate Cox regression analysis based on ATP6V1E1‐related molecules, and functional impacts were assessed through cell proliferation assays. M2 macrophages were the dominant cell type in the HCC TME, significantly correlating with adverse survival outcomes. ATP6V1E1 was robustly associated with advanced disease stages and poor prognostic features such as vascular invasion and elevated alpha‐fetoprotein levels. GSEA linked high ATP6V1E1 expression to critical oncogenic pathways, including immunosuppression and angiogenesis, and reduced activity in metabolic processes like bile acid and fatty acid metabolism. The prognostic model stratified HCC patients into distinct risk categories, showing high predictive accuracy (1‐year AUC = 0.775, 3‐year AUC = 0.709 and 5‐year AUC = 0.791). In vitro assays demonstrated that ATP6V1E1 knockdown markedly inhibited the proliferation of HCC cells. The study underscores the significance of M2 macrophages and ATP6V1E1 in HCC, highlighting their potential as therapeutic and prognostic targets.

## 1 INTRODUCTION

Hepatocellular carcinoma (HCC) is the most common type of primary liver cancer, predominantly affecting individuals with chronic liver diseases, particularly those with hepatitis B, hepatitis C or cirrhosis.^1^, ^2^, ^3^ Globally, HCC is the fourth leading cause of cancer‐related deaths, with particularly high rates in East Asia and sub‐Saharan Africa.^4^ The pathogenesis of HCC is driven by a complex interplay of genetic and environmental factors, including viral infections, excessive alcohol consumption and exposure to aflatoxins.^1^, ^5^ Clinically, HCC often progresses without distinct symptoms in its early stages, which poses significant challenges to early diagnosis.^6^ The current therapeutic landscape for HCC includes options such as surgical resection, liver transplantation, locoregional therapies and systemic approaches like targeted therapy and immunotherapy.^7^ Despite these advancements, the prognosis for advanced HCC remains bleak, highlighting the critical need for early detection and more efficacious treatment modalities.^8^ Recent breakthroughs in molecular biology have paved the way for novel targeted therapies, promising improved outcomes for HCC patients.

M2 macrophages play a pivotal role in creating an immunosuppressive tumour microenvironment that protects cancer cells from immune surveillance and elimination.^9^ By secreting anti‐inflammatory cytokines and growth factors, these macrophages suppress the activity of cytotoxic T cells and natural killer cells—crucial players in the body's anti‐tumour arsenal.^10^ This suppression is especially detrimental in HCC, where it can diminish the effectiveness of emerging immunotherapies. Additionally, M2 macrophages promote angiogenesis by releasing vascular endothelial growth factor (VEGF), which is vital for tumour growth as it supplies the necessary nutrients and oxygen to rapidly proliferating cancer cells.^11^ They also secrete enzymes that degrade the extracellular matrix, facilitating tumour invasion into adjacent tissues and enhancing metastatic potential. Given these functions, targeting M2 macrophages within the HCC microenvironment represents a promising therapeutic strategy.^12^ Approaches aimed at converting M2 macrophages to the pro‐inflammatory M1 phenotype, inhibiting their recruitment or neutralizing their suppressive activities could potentially bolster the immune response, curb tumour progression and significantly improve the prognosis for patients with HCC. Thus, decoding and manipulating the behaviour of M2 macrophages in HCC is paramount to advancing treatment strategies and enhancing patient survival.

This study utilized the CIBERSORT algorithm to analyse the tumour microenvironment in HCC, identifying M2 macrophages as predominant and linked to poor survival. By analysing gene expression, we discovered molecules, notably ATP6V1E1, associated with M2 characteristics and adverse clinical outcomes. Gene Set Enrichment Analysis further implicated ATP6V1E1 in pathways related to tumour progression and immune suppression. Additionally, a prognostic model incorporating ATP6V1E1‐related molecules effectively stratified patients by survival risk. Functional assays confirmed ATP6V1E1's role in promoting HCC cell proliferation, suggesting its potential as a therapeutic target.

## 2 METHODS

**FIGURE 1:**
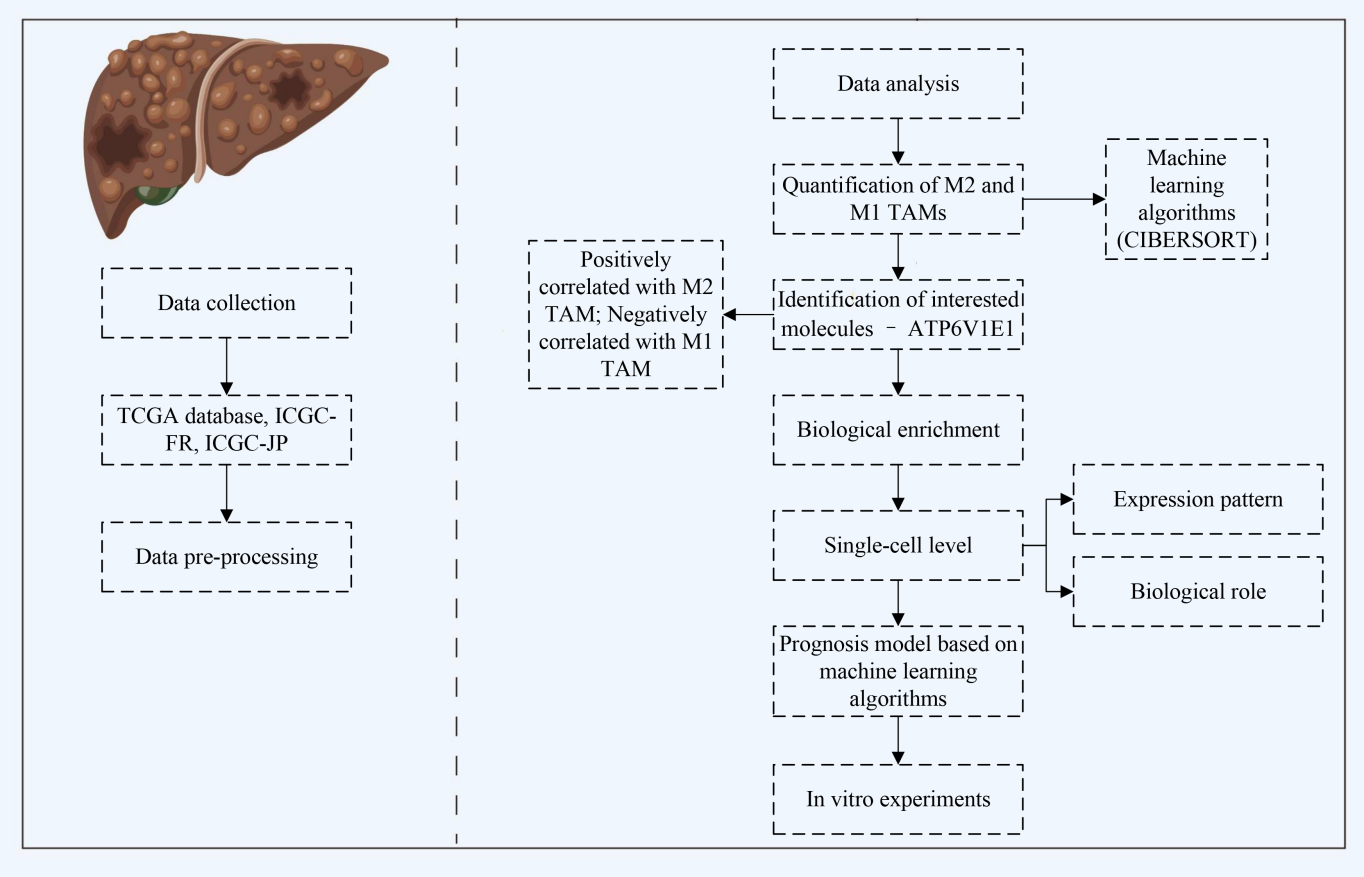
The flowchart of whole study.

The flow chart of the whole study is shown in Figure 1.

### 2.1 Collection and preprocessing of public data

Pan‐cancer expression profiles were downloaded from the UCSC Xena platform (https://xenabrowser.net/datapages/).^13^ For HCC patients, transcription profile data was sourced from The Cancer Genome Atlas (TCGA)—LIHC project and the International Cancer Genome Consortium (ICGC) databases.^14^ TCGA raw expression data, initially in ‘STAR‐Counts’ format, were extracted using custom R scripts and converted into Transcripts Per Million (TPM) units. A human genomic reference file from the Ensemble website was used for gene symbol ID annotation. Preprocessing steps included gene annotation, normalization via log2(TPM + 1) transformation and averaging of duplicated gene measurements. Clinical data were obtained in ‘bcr‐xml’ format and preprocessed for subsequent analyses. Differential gene expression was analysed using the Limma package, adjusting for potential confounders and batch effects.^15^

### 2.2 Quantification of the tumour microenvironment

The tumour microenvironment's cellular composition, with a focus on M2 macrophages, was quantified using the CIBERSORT machine‐learning algorithm.^16^ This involved the analysis of gene expression data to estimate the relative abundance of different immune cell types within the HCC samples.

### 2.3 Biological enrichment

Gene Set Enrichment Analysis (GSEA) was conducted to identify the biological pathways significantly associated with ATP6V1E1 expression and macrophage polarization.^17^ The Hallmark and Kyoto Encyclopedia of Genes and Genomes (KEGG) gene sets were performed using the clusterProfiler package in R, adjusting for multiple testing.

### 2.4 Prognosis analysis

Survival differences were assessed using Kaplan–Meier (KM) survival curves, comparing groups based on macrophage infiltration and ATP6V1E1 expression levels. Prognostic models were constructed using univariate and multivariate Cox regression analyses to identify significant predictors of patient survival. LASSO regression was employed to optimize the selection of prognostic markers by minimizing overfitting.^18^ Model performance was evaluated using receiver operating characteristic (ROC) curves to calculate the area under the curve (AUC) for survival prediction accuracy.

### 2.5 Single‐cell analysis

Single‐cell RNA sequencing data from the TISCH database were analysed to investigate the expression patterns of ATP6V1E1 among different cellular populations within the HCC microenvironment.^19^ Data processing included quality control, normalization and clustering analyses to identify distinct cell types expressing ATP6V1E1.

### 2.6 Cell culture and genetic manipulation

HCC cell lines Huh7 and HepG2, along with the normal liver cell line THLE‐2, were maintained in Dulbecco's Modified Eagle Medium (DMEM) supplemented with 10% fetal bovine serum (FBS), 1% penicillin–streptomycin, at 37°C in a humidified atmosphere containing 5% CO_2_. For genetic manipulations, ATP6V1E1 expression was modulated through plasmid transfection and lentiviral transduction for knockdown studies. Plasmids of shRNA against ATP6V1E1 were transfected into cells using Lipofectamine 2000 according to the manufacturer's protocol. Following transfection, cells were selected with 2 μg/mL puromycin for 2 weeks to establish stable cell lines expressing knocked‐down ATP6V1E1.

### 2.7 Quantitative real‐time PCR (qPCR)

Total RNA was extracted from the cultured cell lines using TRIzol reagent according to the manufacturer's instructions. The concentration and purity of RNA were assessed using a NanoDrop spectrophotometer, and RNA integrity was verified via agarose gel electrophoresis. Complementary DNA (cDNA) was synthesized from 1 μg of total RNA using the High‐Capacity cDNA Reverse Transcription Kit with random primers. Real‐time PCR was then performed using SYBR Green PCR Master Mix on a QuantStudio 5 Real‐Time PCR System. Specific primer sequences for ATP6V1E1 were as follows: forward, 5′‐AACATAGAGAAAGGTCGGCTTG‐3′ and reverse, 5′‐GACTTTGAGTCTCGCTTGATTCA‐3′. The housekeeping gene GAPDH was used as an internal control with primers: forward, 5′‐GGAGCGAGATCCCTCCAAAAT‐3′ and reverse, 5′‐GGCTGTTGTCATACTTCTCATGG‐3′.

### 2.8 Cell proliferation assays

The proliferative capacity of HCC cell lines following modulation of ATP6V1E1 expression was quantitatively assessed using two methods: the Cell Counting Kit‐8 (CCK‐8) and colony formation assays. For the CCK‐8 assay, cells were seeded in 96‐well plates at a density of 2000 cells per well and allowed to adhere overnight. Post‐transfection (24, 48 and 72 h), CCK‐8 solution was added to each well, and cells were incubated for an additional 2 h. Absorbance was measured at 450 nm using a microplate reader to determine cell viability. For the colony formation assay, cells were plated at a low density (500 cells per well) in 6‐well plates and cultured for 2 weeks. Colonies were fixed with methanol, stained with 0.1% crystal violet and counted manually. Each assay was performed in triplicate to ensure reproducibility.

### 2.9 Statistical analysis

All statistical analyses were performed using R software. Data were tested for normality and homogeneity of variance to select appropriate statistical tests, including *t*‐tests, ANOVA or non‐parametric equivalents, depending on the data distribution.

## 3 RESULTS

### 3.1 Identification of M2 macrophage‐related molecules using machine learning algorithms

**FIGURE 2:**
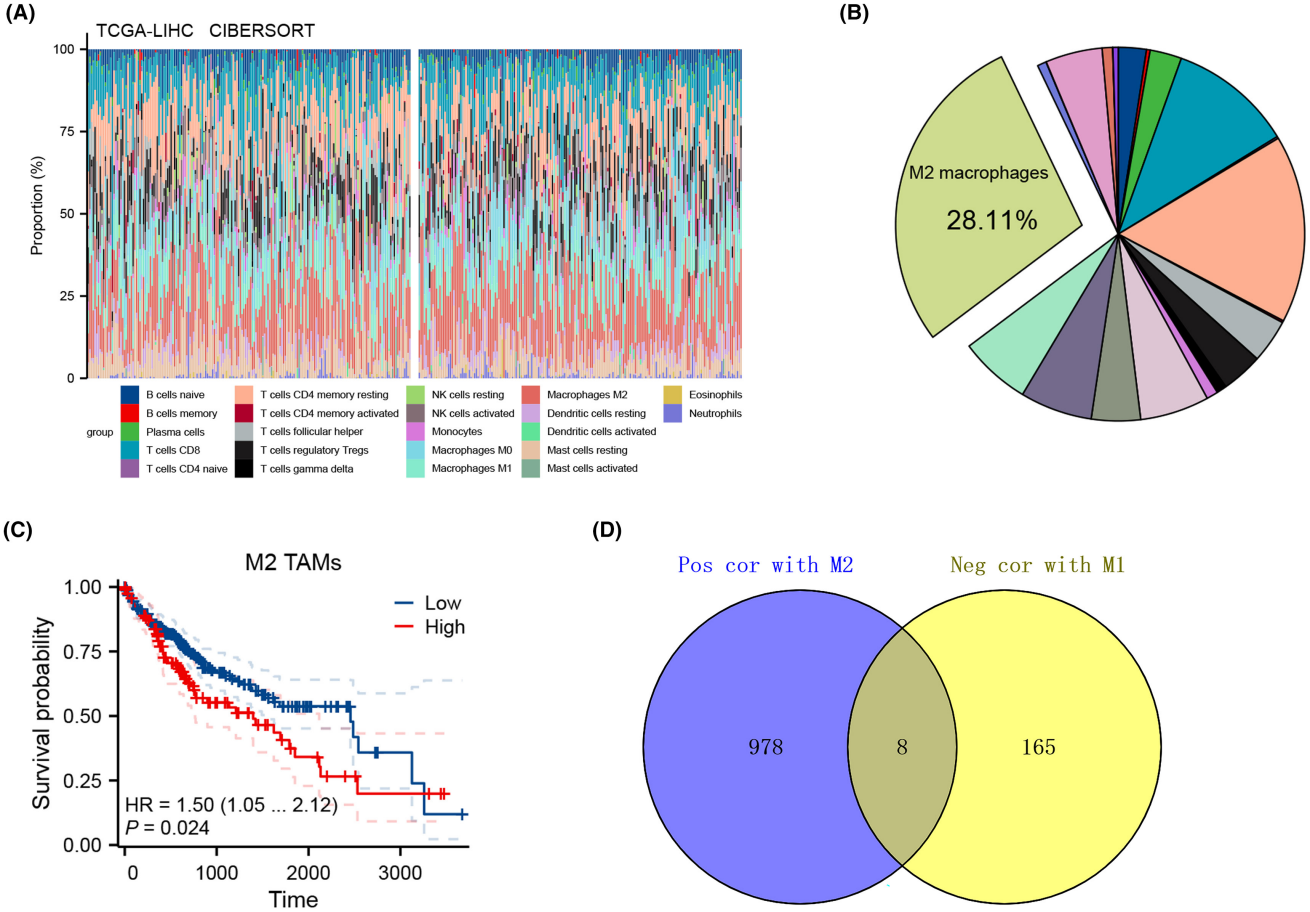
Analysis of M2 Macrophage infiltration and associated molecular profiles in HCC. (A) Bar graph depicting the overall cellular composition of the HCC tumour microenvironment, quantified using the CIBERSORT algorithm; (B) Pie chart demonstrating the percentage of M2 macrophages in the tumour microenvironment, establishing them as the predominant cell type at 28.11% of the total immune cell population; (C) KM survival curves displaying the relationship between M2 macrophage infiltration levels and patient survival outcomes. The graph distinctly shows that higher infiltration of M2 macrophages correlates with poorer survival, emphasizing their impact on HCC prognosis; (D) Venn plot identifying specific molecules that are positively correlated with M2 macrophage levels and negatively correlated with M1 macrophages in the tumour microenvironment. Molecules such as TTC39A, HPGDS, MSX1, CYCS, FJX1, ATP6V1E1, RAB6B and NDRG4 are marked for their significant associations, which may represent potential therapeutic targets or biomarkers.

Utilizing the CIBERSORT algorithm, we quantified the cellular composition of the tumour microenvironment in HCC (Figure 2A). Notably, M2 macrophages emerged as the predominant cell type, accounting for 28.11% of the cellular milieu, underscoring their critical role in HCC (Figure 2B). KM survival analysis revealed that higher M2 macrophage infiltration correlates with poorer survival outcomes in patients (Figure 2C). To further dissect this relationship, we identified molecules positively associated with M2 macrophage levels and inversely associated with M1 macrophages. Eight molecules met our stringent criteria: TTC39A, HPGDS, MSX1, CYCS, FJX1, ATP6V1E1, RAB6B and NDRG4 (Figure 2D).

### 3.2 Clinical correlation of ATP6V1E1

**FIGURE 3:**
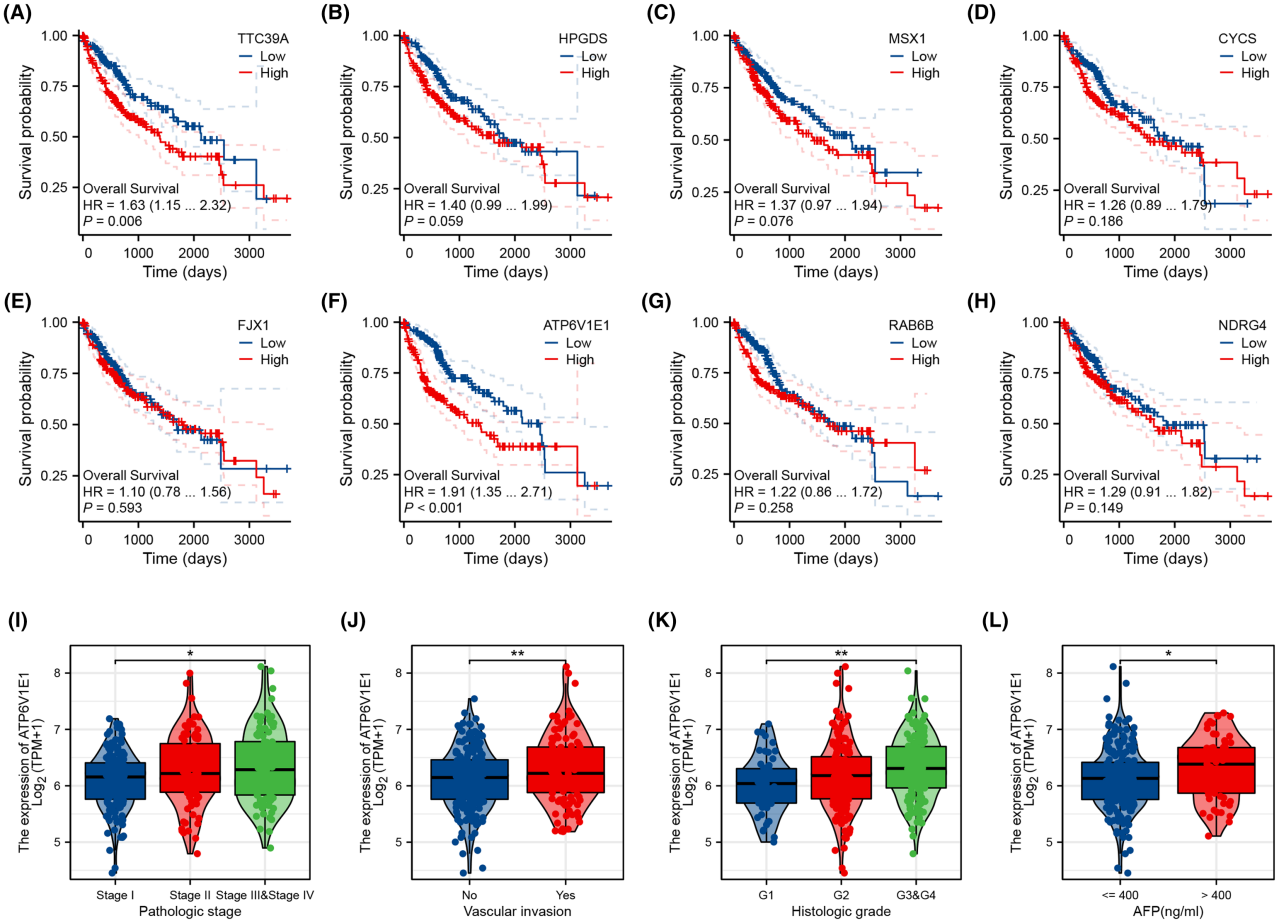
Clinical correlation of ATP6V1E1 and TTC39A in HCC. (A–H) KM survival curves illustrating the patients with higher expression levels of TTC39A and ATP6V1E1 might have shorter survival times, suggesting their utility as prognostic markers; (I–L) Box plots depicting the relationship between ATP6V1E1 expression and various clinical features including advanced pathological stages, vascular invasion, histologic grades and AFP levels. These plots demonstrate a strong association of high ATP6V1E1 expression with aggressive disease features.

Further investigations into the prognostic significance of these molecules demonstrated that high expression levels of TTC39A and ATP6V1E1 are predictive of adverse survival outcomes (Figure 3A–H). Given the pronounced statistical significance of ATP6V1E1 (lowest *p*‐value), we prioritized it for deeper clinical correlations. Our analysis linked higher ATP6V1E1 expression with advanced pathological stages, vascular invasion, higher histologic grades and elevated AFP levels, implicating its association with aggressive disease features (Figure 3I–L).

### 3.3 Biological enrichment analysis of ATP6V1E1

**FIGURE 4:**
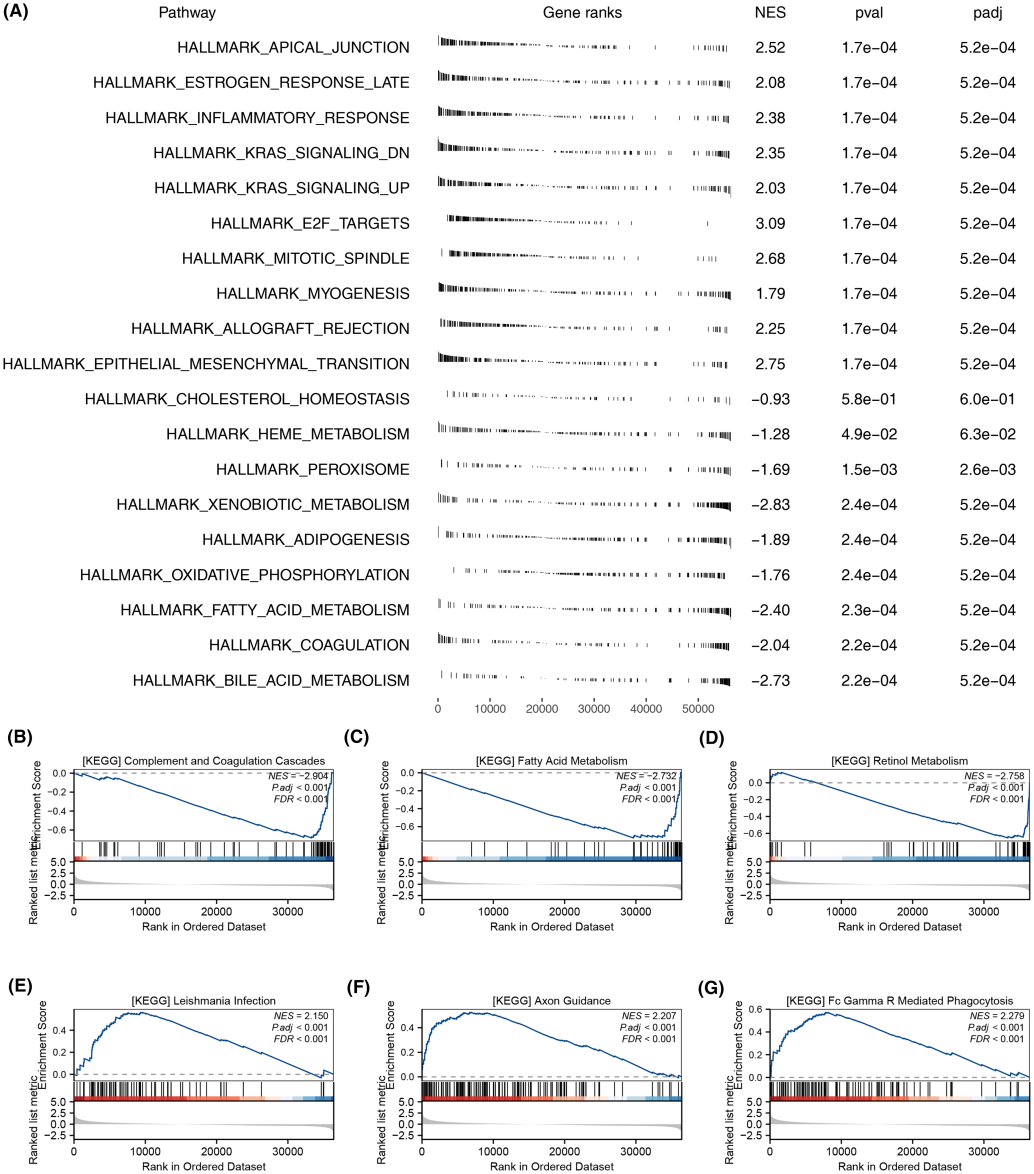
Biological enrichment analysis of ATP6V1E1. (A) GSEA analysis of ATP6V1E1 based on Hallmark gene set; (B–G) GSEA analysis of ATP6V1E1 based on KEGG gene set.

GSEA using the Hallmark gene set revealed enhanced activity in pathways such as apical junction, oestrogen response late and inflammatory response in patients with elevated ATP6V1E1 expression. Conversely, these patients showed reduced activity in bile acid metabolism, coagulation and fatty acid metabolism (Figure 4A). Similarly, GSEA with the KEGG gene set indicated increased activity in leishmania infection, axon guidance and Fc gamma R‐mediated phagocytosis pathways, whereas activities of complement and coagulation cascades, fatty acid and retinol metabolism were diminished (Figure 4B–G).

### 3.4 Single‐cell analysis of ATP6V1E1 in the HCC microenvironment

**FIGURE 5:**
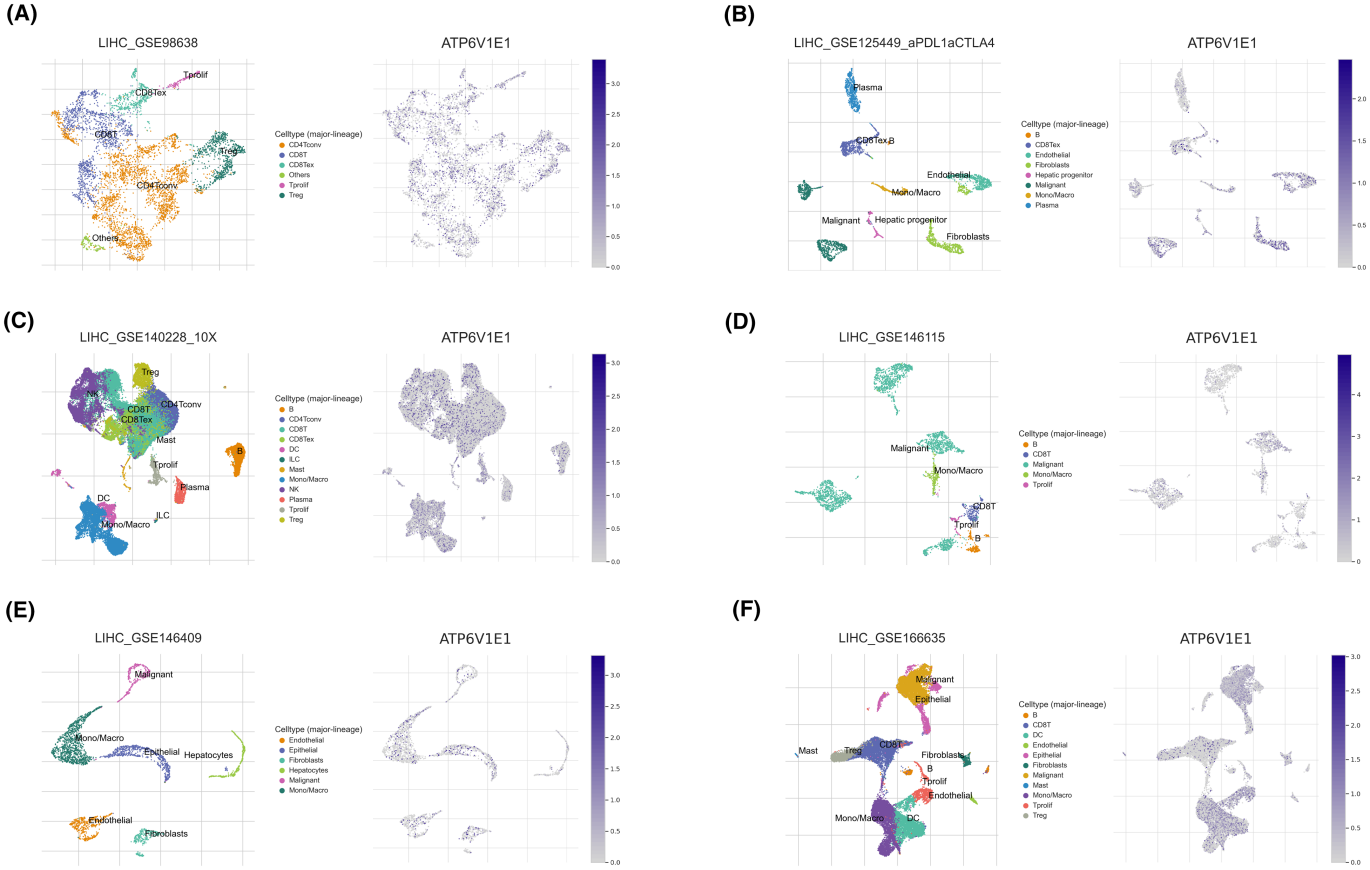
Single‐cell analysis of ATP6V1E1 in the HCC microenvironment. (A–F) Composite of scatter and density plots showing the distribution and expression levels of ATP6V1E1 across different cellular populations within the HCC tumour microenvironment.

**FIGURE 6:**
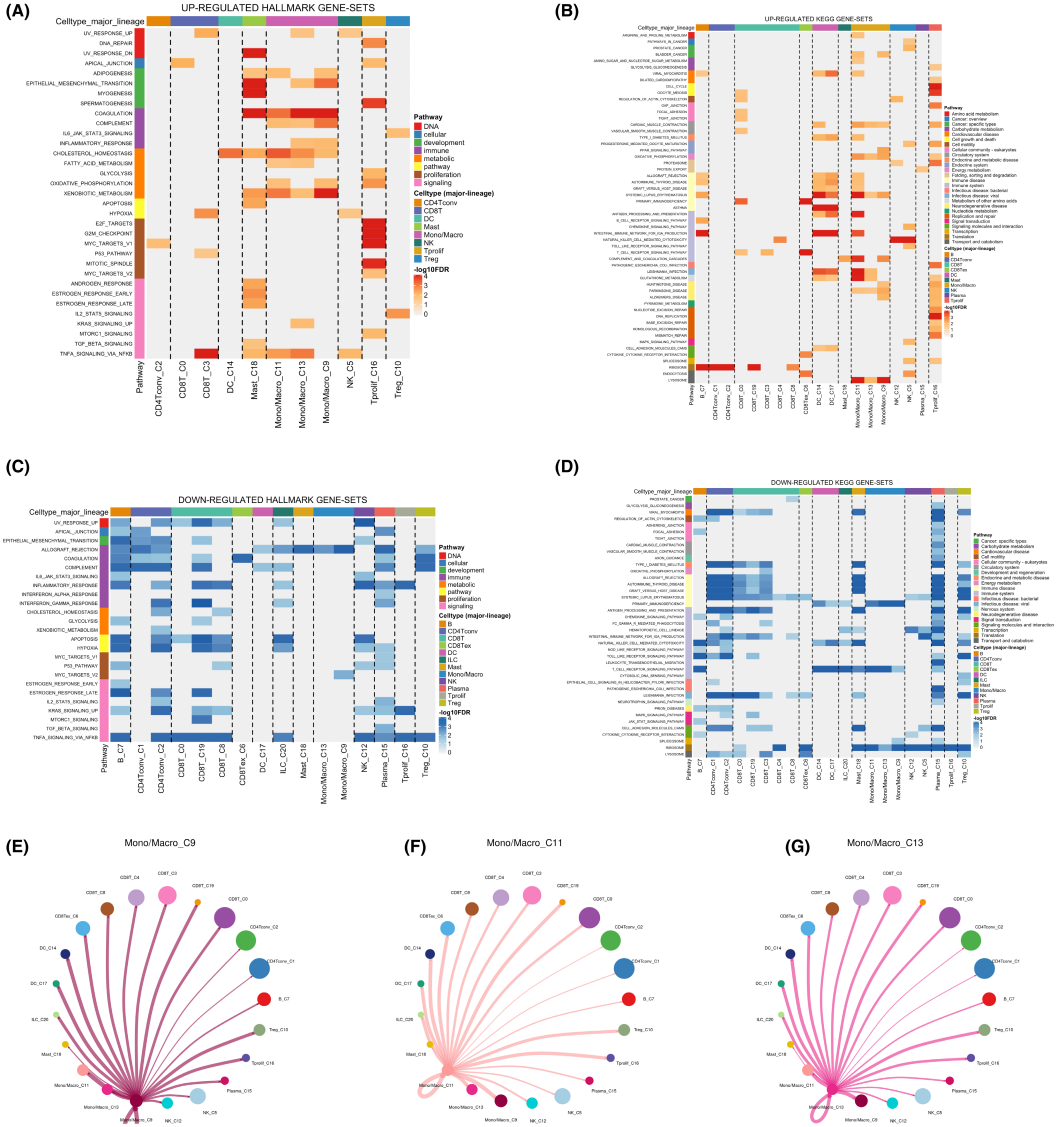
Macrophage functions and cell interactions in HCC. (A–D) The correlation between macrophage activity and various metabolic and immune pathways, specifically illustrating positive associations with coagulation, complement cascades, cholesterol homeostasis and xenobiotic metabolism, and a negative correlation with allograft rejection; (E–G) Visualizations of cell–cell interactions within the HCC microenvironment.

At the single‐cell level, ATP6V1E1 was ubiquitously expressed across diverse cellular populations within the HCC microenvironment (Figure 5A–F). Given its strong correlation with M2 macrophages, we explored macrophage functions, revealing their positive associations with coagulation, complement cascades, cholesterol homeostasis and xenobiotic metabolism. However, macrophages showed a negative correlation with allograft rejection (Figure 6A–D). Cell interaction studies highlighted predominant interactions between macrophages and T cells, further delineating their role in the immune landscape of HCC (Figure 6E–G).

### 3.5 Prognostic model based on ATP6V1E1‐related molecules

**FIGURE 7:**
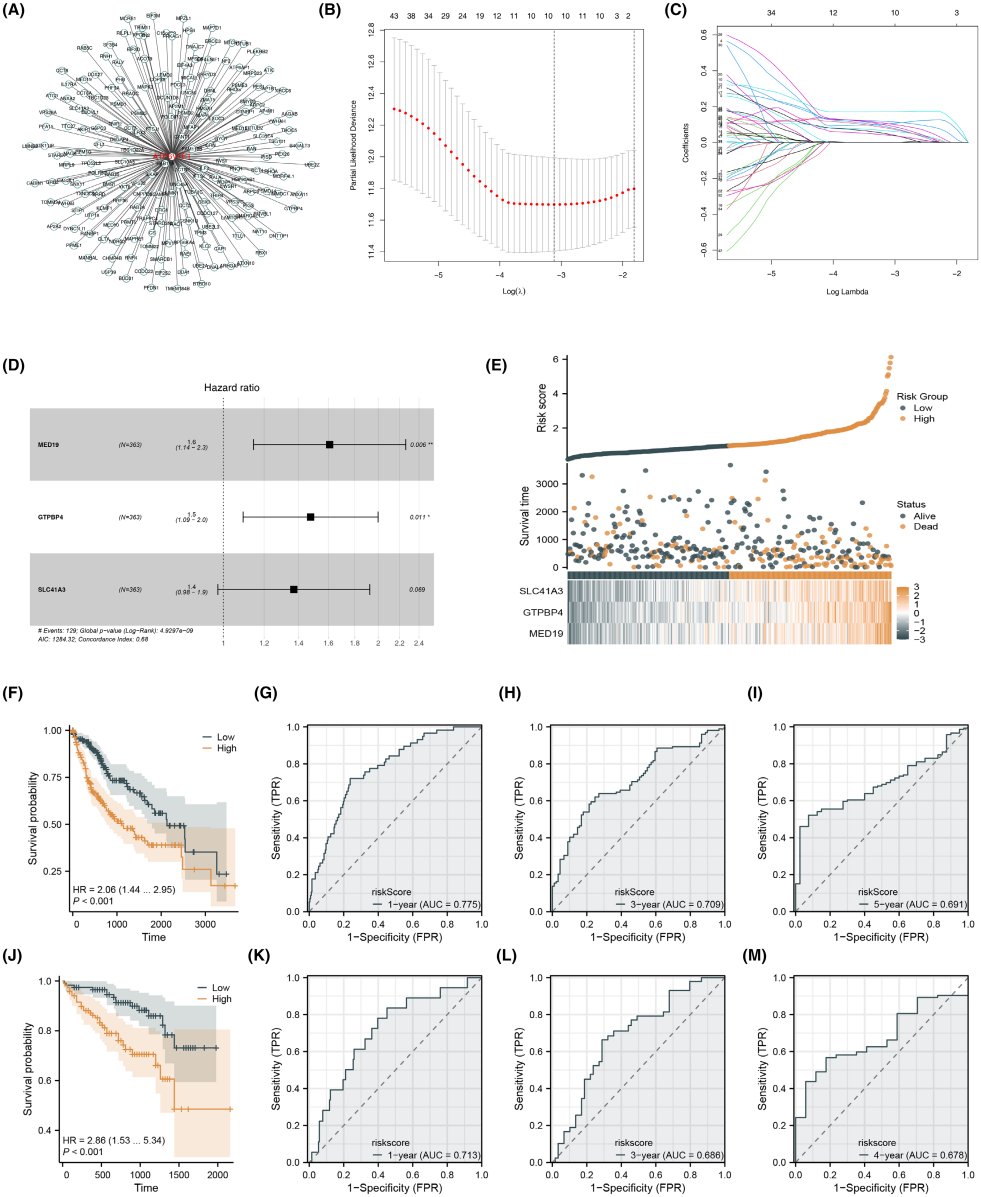
Prognostic model based on ATP6V1E1‐related molecules. (A) The molecules with significant correlation with ATP6V1E1; (B, C) LASSO regression analysis; (D, E) Multivariate Cox regression outputs, culminating in a risk score formula; (F–I) Validation of risk score through KM and ROC curves; (J–M) Additional analysis in the ICGC cohort demonstrate the model's efficacy and robust predictive accuracy.

To enhance the clinical applicability of our findings, we performed a correlation analysis identifying 200 molecules strongly associated with ATP6V1E1 expression (Figure 7A and Appendix S1). Subsequent univariate Cox regression analysis in the TCGA cohort identified 50 molecules with significant survival impact (Appendix S2), which served as inputs for LASSO regression to reduce dimensionality (Figure 7B,C). Multivariate Cox regression highlighted key prognostic molecules—MED19, GTPBP4 and SLC41A3 for inclusion in our risk score formula, which significantly stratified patients into high‐ and low‐risk groups (risk score = MED19 * 0.476 + GTPBP4 * 0.390 + SLC41A3 * 0.315, Figure 7D–F). The risk score model demonstrated robust predictive accuracy in survival outcomes, confirmed through ROC analysis across multiple time points (1‐year AUC = 0.775, 3‐year AUC = 0.709 and 5‐year AUC = 0.791). Validation in the ICGC cohort reinforced these findings (Figure 7G–M).

### 3.6 Role of ATP6V1E1 in HCC cell proliferation

**FIGURE 8:**
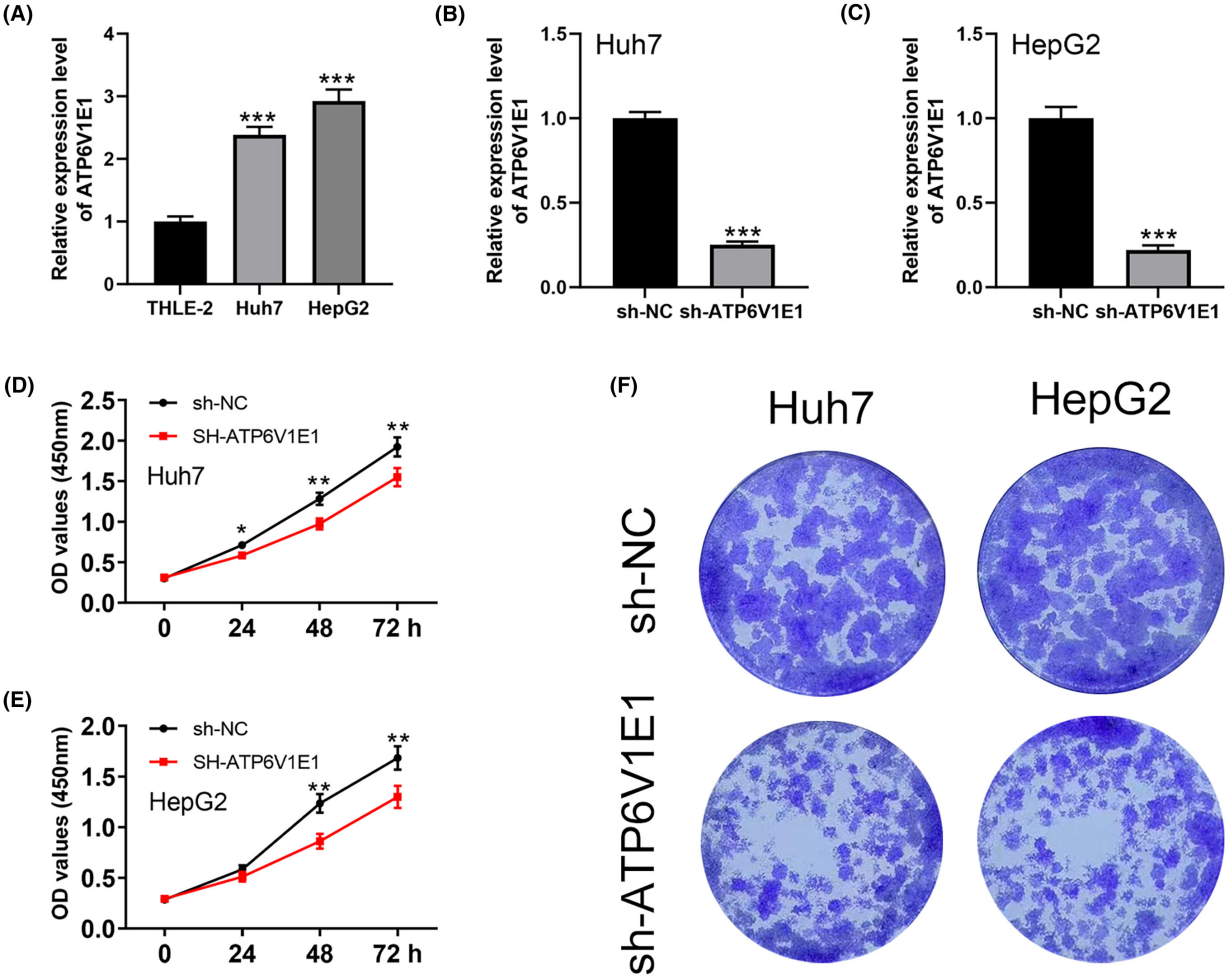
Role of ATP6V1E1 in HCC cell proliferation. (A) Comparative analysis graphs showing ATP6V1E1 expression levels in HCC cell lines (Huh7, HepG2) versus normal liver cells (THLE‐2), indicating significant upregulation in tumour cells; (B, C) The knockdown efficiency of ATP6V1E1 in HCC cell lines; (D–F) Results from functional assays including CCK‐8 and colony formation tests, which confirm that silencing ATP6V1E1 markedly inhibits the proliferative capacity of HCC cells.

Comparative analysis of ATP6V1E1 expression in HCC cell lines (Huh7, HepG2) versus normal liver cells (THLE‐2) showed significant upregulation in HCC cells (Figure 8A–C). Functional assays, including CCK‐8 and colony formation tests, confirmed that silencing ATP6V1E1 markedly reduced the proliferative capacity of HCC cells, highlighting its potential as a therapeutic target (Figure 8D–F).

## 4 DISCUSSION

The findings from this study elucidate the complex interplay between the tumour microenvironment and HCC progression, with a specific focus on the role of M2 macrophages and the molecular pathways influenced by ATP6V1E1. The predominance of M2 macrophages within the TME of HCC patients, as identified through CIBERSORT analysis, underscores their significant role in tumour dynamics and their potential as targets for therapeutic intervention.

M2 macrophages are known for their immunosuppressive functions, promoting tumour growth and survival by inhibiting cytotoxic T cell activity and supporting angiogenesis and tissue remodelling.^20^, ^21^ The substantial presence of these macrophages in HCC, correlating with poor patient survival, aligns with previous studies that highlight the tumour‐promoting capabilities of M2 macrophages in various cancers.^22^, ^23^ For example, Hao et al. performed deep single‐cell RNA sequencing on immune cells from HCC patients and controls, identifying distinct subtypes and pathways, and demonstrated that inhibiting APOC1 can shift macrophages from the M2 to the M1 phenotype via the ferroptosis pathway, potentially improving the efficacy of anti‐PD1 immunotherapy in HCC. This study extends that knowledge by quantifying their prevalence and correlating it with clinical outcomes, emphasizing the need for strategies that could modulate macrophage polarization as a therapeutic approach.

The ATP6V1E1 gene encodes a protein known as ATP6V1E1, which is a subunit of the V‐ATPase (vacuolar‐type H + ‐ATPase) complex.^24^ V‐ATPase is a multi‐subunit enzyme primarily located in the lysosomes and endosomes of cells, responsible for transporting protons from the cytoplasm into these organelles, thereby maintaining their acidic environment.^25^ ATP6V1E1 plays a crucial role in this process by participating in proton translocation across membranes, regulating intracellular pH.^26^ In addition to its fundamental role in cellular homeostasis, ATP6V1E1 has been implicated in various diseases.^27^ For example, dysregulation of V‐ATPase activity, including the ATP6V1E1 subunit, is associated with cancer progression. The acidic microenvironment created by V‐ATPase is conducive to tumour invasion, metastasis and resistance to chemotherapy.^27^ Furthermore, alterations in ATP6V1E1 expression have been linked to neurodegenerative diseases, where impaired lysosomal function leads to the accumulation of toxic proteins and cellular debris.^28^ Therefore, the ATP6V1E1 gene and its encoded protein are of significant importance in cellular biology research and disease mechanism exploration.

The association of ATP6V1E1 with adverse features such as advanced pathological stages, vascular invasion and high histologic grades, as well as elevated alpha‐fetoprotein levels, points to its role in facilitating aggressive HCC behaviour. GSEA provided deeper insights into the biological functions of ATP6V1E1, linking high expression to enhanced activity in pathways associated with apical junction, oestrogen response and inflammatory response, while showing decreased activity in pathways like bile acid metabolism, coagulation and fatty acid metabolism. Such findings suggest that ATP6V1E1 may be involved in remodelling the cellular environment to favour tumour survival and progression, possibly through mechanisms that enhance cellular proliferation and resistance to apoptosis.^29^ Given the immunosuppressive nature of M2 macrophages in HCC, the current findings highlight a significant barrier to the efficacy of immunotherapeutic approaches.^30^ The interaction between ATP6V1E1 expression and macrophage‐mediated immune suppression could explain some of the variability in patient responses to immunotherapy. Targeting ATP6V1E1 or modulating macrophage polarization might enhance the effectiveness of these therapies by shifting the immune landscape of the TME from a suppressive to a more active state, thereby improving patient outcomes.

The prognostic model developed using ATP6V1E1‐related molecules provides a new tool for stratifying HCC patients into risk categories, which could potentially guide clinical decision‐making. This model's ability to predict survival outcomes, as demonstrated through robust ROC analysis, underscores its utility in clinical settings, offering a method to personalize therapeutic approaches based on molecular profiling. The functional assays performed in this study demonstrate that ATP6V1E1 not only serves as a biomarker of poor prognosis but also actively contributes to the oncogenic processes in HCC. By showing that silencing ATP6V1E1 markedly reduces the proliferative capacity of HCC cells, these results support the rationale for developing ATP6V1E1 inhibitors as potential therapeutic agents.

One of the main limitations of this study is its retrospective nature, relying on existing data from databases like TCGA and ICGC, which may introduce selection bias or incomplete data issues. Additionally, the functional roles of ATP6V1E1 identified through GSEA require experimental validation to confirm causality. The study's reliance on computational predictions and in vitro results also necessitates cautious interpretation before clinical application. Moreover, while the study provides a prognostic model and identifies potential therapeutic targets, translating these findings into effective therapies requires overcoming significant hurdles, including drug design, delivery challenges and the management of potential side effects.

In conclusion, while this study advances our understanding of the roles of M2 macrophages and ATP6V1E1 in HCC progression and provides a foundation for new therapeutic approaches, significant work remains. Addressing the limitations noted and extending the research into preclinical and clinical trials will be crucial for harnessing the full therapeutic potential of these findings. As we continue to unravel the complexities of the TME, integrating these insights into clinical practice could dramatically alter the therapeutic landscape for HCC.

## AUTHOR CONTRIBUTIONS

**Sen Zhao:** Conceptualization (equal); data curation (equal); formal analysis (equal); methodology (equal); software (equal); supervision (equal). **Meimei Liu:** Formal analysis (equal); investigation (equal); methodology (equal); resources (equal); software (equal); validation (equal). **Hua Zhou:** Conceptualization (equal); data curation (equal); investigation (equal); methodology (equal); resources (equal); software (equal); supervision (equal).

## FUNDING INFORMATION

This study was funded by Key Project of Philosophy and Social Science Research in Anhui Province Universities (2023AH052580); Key Natural Science Research Projects in Anhui Province (2023AH052599); Key Natural Science Research Projects in Anhui Province (KJ2021A1272).

## CONFLICT OF INTEREST STATEMENT

The author reports no conflicts of interest in this work.

## Supporting information


Appendix S1.

Appendix S2.

## Data Availability

The datasets used and/or analyzed during the current study are available from the corresponding author on reasonable request.
